# Clinical consequences of untreated dental caries assessed using PUFA index and its covariates in children residing in orphanages of Pakistan

**DOI:** 10.1186/s12903-017-0399-9

**Published:** 2017-07-11

**Authors:** Ramsha Kamran, Warda Farooq, Mehreen Riaz Faisal, Faisal Jahangir

**Affiliations:** 1 Margalla Institute of Health Sciences, Quaid-e-Azam Avenue, Gulrez III, Rawalpindi, Pakistan; 2 Department of Community & Preventive Dentistry, Margalla Institute of Health Sciences, Quaid-e-Azam Avenue, Gulrez III, Rawalpindi, Pakistan; 3 Department of Oral Medicine, Margalla Institute of Health Sciences, Quaid-e-Azam Avenue, Gulrez III, Rawalpindi, Pakistan

**Keywords:** Untreated dental caries, Orphanage children, Pufa index, Decayed extracted filled index

## Abstract

**Background:**

The purpose of this study was to determine the prevalence and clinical effects of untreated dental caries in Pakistani children residing in orphanages using the DMFT and PUFA index; association of decay and untreated dental caries with demographics including type of orphanage; behavioural and dental visiting pattern; and association of dental pain experience and type of orphanage with dental visiting.

**Methods:**

A cross-sectional survey was conducted on a total of 753 orphan children belonging to 4-17 years of age group residing in twin cities of Rawalpindi and Islamabad, Pakistan. Clinical examination of children was performed using the DMFT and PUFA index for the assessment of dental caries and untreated decay, followed by questionnaire enquiring about eating and oral hygiene habits, dental visiting pattern and dental pain and swelling experience. Association between dental decay, child’s dental visiting and pain as a consequence of untreated decay was carried out using chi square test and logistic regression analysis.

**Results:**

The overall caries prevalence was 34.8% and overall prevalence of PUFA/pufa was 15.9%. The mean score of DMFT and dmft was 1.18 (SD 0.39) and 1.04 (SD 0.23), and mean PUFA was 1.18 (SD 0.57) and mean pufa score 1.14 (SD 0.35). Untreated caries ratio was found to be 49.1% indicating half the decay had progressed to involve the pulp. No significant association of gender was found with DMFT, dmft, PUFA and pufa (*p* > 0.05), however, when analysed individually, the ‘D’ component of DMFT was significantly associated with male gender (*p* = 0.05). Furthermore, no significant association of DMFT/dmft or PUFA/pufa in either dentition was found with behavioural characteristics such as dietary and oral hygiene habits. Also, 66.2% children who experienced pain had not been to the dentist in the past year (*p* = 0.013) and 52.6% children who mentioned experiencing pain at night had not been to the dentist in the past year (*p* = 0.009). Children with decay were more likely to have visited the dentist (OR 3.3, 95% CI 1.42-7.6, *p* = 0.006). However, children who reported to have experienced pain were less likely to have visited the dentist in the past year (OR 0.53, 95% CI 0.32-0.88, *p* = 0.014).

**Conclusions:**

Moderate levels of decay were found in the sample with ‘d’ component majorly responsible for the cumulative DMFT index. However, alarmingly almost half of the decay component (49.1%) had progressed to involve the pulp. Experiencing pain in teeth prompted dental visits. Initiation of preventive services for children residing in orphanages in Pakistan would help greatly towards reducing the burden of untreated decay.

**Electronic supplementary material:**

The online version of this article (doi:10.1186/s12903-017-0399-9) contains supplementary material, which is available to authorized users.

## Background

Dental decay is a major frequent non-communicable form of disease that affects 60-90% children worldwide [1]. More recently, Marcenes et al. reported untreated decay of the permanent teeth as the most prevalent condition world over and untreated decay of deciduous dentition 10th most prevalent condition, in the Global Burden of Disease Study, 2010 [2]. Untreated dental caries can adversely affect children’s quality of life and result in severe consequences including risk of dental sepsis [3]. A significant association has also been reported between untreated decay and low body mass [4].

Orphans are particularly at risk of untreated decay due to lack of parental support and neglected oral health care [5]. UNICEF, UNAIDS & USAID reported that orphanages can be unfavourable for a child’s growth and development because for children to survive and thrive, they need to grow up in a community and family environment that caters to their changing needs. Development of children to their full potential is seriously hampered due to deterioration of family environment [6]. As a result a high prevalence of dental caries [7], dental trauma and gingivitis is seen in children from orphanages [8].

In Pakistan, dental caries have been reported to be the most prevalent childhood disease, 5 times more prevalent than Asthma and 7 times more common than Hayfever [9]. Caries prevalence of 71.7% has been reported in 6-14 year old children in Karachi [10]. Similar levels of decay have been seen in Peshawar (72.4%) in school going children of class one to six [11]. In Sargodha district of Pakistan, a study reported caries prevalence of 45.9% in 3-12 year old children [12], and In Rawalpindi and Islamabad, caries prevalence in 5 year old children has been reported to be 44.4% [13].

The caries prevalence in institutionalized children world over has been reported to be higher than those children who live with their families. Prevalence of decay in orphanage children in India has been reported to be 88.5% [14]. Similar decay levels have been reported in institutionalized 12-15 years old adolescents in Yemen (84.7%) [15] and 4-12 year old orphans in Saudi Arabia (96%) [16]. In China, 50% decay levels have been reported in 4-17 year old orphan children and adolescents [17].

Dental caries is a major community health problem and many studies exist which document the burden of untreated decay, yet data available on its clinical sequel is limited [18–20]. DMFT/dmft index has been used worldwide to collect data on dental caries for so many decades, however, this cumulative index does not provide information on the clinical consequences of untreated dental caries, such as involvement of the pulp and development of abscess, which may be more jeopardous than the lesion itself, but rather provides information on its surgical and restorative treatment needs. To determine the severity and extensiveness of oral conditions that result due to untreated dental caries, index by the name of PUFA was presented by Monse et al. [21]. When used in conjunction with DMFT index, it helps in projecting the clinical consequences of untreated carious lesion [22]. The PUFA Index records the presence of grossly decayed teeth with, P/p: visible pulpal involvement, U/u: ulceration due to trauma from tooth fragments, F/f: fistula and A/a: abscess. Uppercase letters are for permanent and lowercase letters are for the deciduous teeth. Teeth that fulfil the PUFA diagnostic standard are scored and estimated cumulatively. Data collected through this index can have impact on decision taken by dental practitioners and decision makers, which cannot be obtained by DMFT index alone [23].

Not much work has been done in Pakistan to report the clinical consequences of untreated decay. As there is very limited data available about oral health status of socially deprived children residing in orphanages in Pakistan, the aim of our study is to report the clinical consequences of untreated decay in children living in orphanages of the twin cities of Rawalpindi and Islamabad, and the association of decay and untreated dental caries with demographics including type of orphanage, behavioural, and dental visiting pattern; and association of dental pain experience and type of orphanage with dental visiting.

## Methods

A cross-sectional survey was conducted on a total of 753 orphan children aged 4-17 years residing in orphanages in twin cities of Rawalpindi and Islamabad, Pakistan. The minimum required sample size calculated for this study was 384 with 95% confidence Interval and 5% margin of error.

Both the cities of Rawalpindi and Islamabad are major cities of Pakistan and are collectively known as the ‘Twin cities’ because they lie side by side, just a mere 15 km distance apart. The Rawalpindi-Islamabad Metropolitan area is the third largest in Pakistan with a population exceeding five million (Pakistan Bureau of statistics). The twin cities house a number of orphanages both private and government owned, however, no data on their oral health status is available.

The study adopted multistage random sampling technique in which from the list of orphanages of the twin cities, random selection was done on the basis of type of ownership of the orphanage (government or private) through a computer generated sequence. Hence, one government and one privately owned orphanage was randomly selected from each of the Twin cities. The inclusion criteria was children and adolescents aged 4-17 years of both genders who agreed to participate in the study. To avoid incorporation of bias, children with physical or mental disability, and any developmental dental anomaly were not included in the analysis because children with such conditions have difficulty in managing routine oral hygiene measures and hence are more prone to dental caries.

Ethical approval was obtained from Institute’s Research and Ethics committee. A meeting was then arranged with the primary care giver of the orphanages and after having explained to them the aims and objectives of the study, their written consent was obtained. The children were also informed about the study including the benefits and risks and were given the option to opt out if they were not willing to participate.

Data collection consisted of clinical examination and answering a questionnaire. Two trained dentists carried out the clinical examination of children according to the WHO methodology (1997), under the supervision of a senior dentist [24]. Since this study was part of a larger study, the examiners were trained and calibrated before the start of the whole project by a senior dentist with considerable experience in clinical dentistry. Reliability analysis were undertaken by examining 15 children not included in this study. The Intraclass correlation coefficient showed excellent agreement between both the examiners for dmft (0.93) as well as pufa (0.80).

Children were clinically examined in their respective orphanage, seated on a normal chair in daylight. The assessment was made visually without the use of any instrument except mouth mirror and gauze pads. Gauze pads were used only to remove food debris from the surface of the teeth. Barriers like gloves and masks were also used for personal protection. Prevalence of dental caries was scored by using DMFT/dmft index following the WHO criteria [24].

PUFA Index: This index was recorded separately from the DMFT index and was used to score the presence of PUFA according to the following codes and criteria as mentioned by the developers:

Only one score is assigned per tooth. When in doubt concerning the extent of odontogenic infection, the basic score (P/p for pulp involvement) is given. If the primary tooth and its permanent successor tooth are present and both show stages of odontogenic infections, both teeth will be scored. Upper case letters are used for the permanent dentition and lower case for the primary dentition. The PUFA score per person is calculated in the same cumulative way as for the dmft and represents the number of teeth that meet the PUFA diagnostic criteria. The PUFA for permanent teeth and primary teeth are reported separately. Thus, for an individual person the score ranges from 0 to 20 pufa for the primary dentition and from 0 to 32 PUFA for the permanent dentition.

A structured questionnaire was prepared to evaluate and record the oral hygiene practice, dietary habits and history of dental pain experience and dental visiting of the orphanage children (Additional file 1: Dental Questionnaire). The questionnaire comprised of 12 questions and was developed by the authors keeping in view the cultural context of the study. First two questions were about the individual’s oral hygiene practice such as tooth brushing frequency and the tooth cleaning method used. These two questions were adapted from the questionnaire used in study conducted by Shahbong et al. in orphanage children in India [14]. The next two questions enquired about the frequency of food/snacks taken per day and fizzy/carbonated drinks consumed during a week. Rest of the questions were related to frequency of dental visits, and problems with teeth and gums including episode and location of pain. Children were informed about how to fill the questionnaire and to ask for help if they faced any difficult in answering it. Children less than 10 years of age, were helped with filling the questionnaires by reading out the questions and explaining to them in local language to elicit their answer.

Data analysis was carried out using SPSS version 21. Caries prevalence and overall PUFA/pufa prevalence was calculated as percentage. Mean DMFT score for both primary and permanent dentition was reported. Untreated dental caries severity was recorded using PUFA index and reported as mean score. Untreated caries-pufa ratio calculated using [PUFA + pufa/D + d] × 100, provided information about percentage of teeth with untreated caries that developed odontogenic infection [21]. Chi square test was used to study association between categorical data such as age, gender, type or orphanage, dental visiting and pain, dmft/DMFT and pufa/PUFA. A binary logistic regression model was fitted to determine the effect of decay and pain on dental visiting.

## Results

Total of 753 children were examined. Out of the total, majority were males (72.1%) and 27.9% were females. Children ages ranged from 4 to 17 years with mean age of 10.5 years. All the children present in the orphanage on the day examination agreed to take part in the study.

The mean score of dmft was 1.04 (SD 0.23) and DMFT was 1.18 (SD 0.39). A very small ‘f’ component was found in both the dentition. The mean pufa was 1.18 (0.57) and mean PUFA 1.14 (SD 0.35). No fistula and abscess were found for permanent dentition, however, abscess was reported with 2 teeth in primary dentition (Table 1).

**Table 1:** Demographic details and DMFT/dmft and PUFA/pufa score of primary and permanent dentition of 753 children

Characteristic	*N* (%)
Gender	
Male	543 (72.1)
Female	210 (27.9)
Age (years)	
4-5	25 (3.3)
6-12	528 (70.1)
13-17	200 (26.5)
Permanent dentition	Mean (SD)
Decay (D)	0.83 (0.38)
Missing (M)	0.16 (0.37)
Filled (F)	0.006 (0.08)
DMFT	1.18 (0.39)
Pus (P)	0.86 (0.35)
Ulceration (U)	0.86 (0.35)
Fistula (F)	0
Abscess (A)	0
PUFA	1.14 (0.35)
Primary dentition	Mean (SD)
Decay (d)	0.97 (0.17)
Missing (m)	0.02 (0.15)
Filled (f)	0.007 (0.09)
dmft	1.04 (0.23)
pus (p)	0.88 (0.33)
ulcer (u)	0.09 (0.29)
fistula (f)	0
abscess (a)	0.09 (0.29)
pufa	1.18 (0.57)

No association of dmft (*p* = 0.29) and DMFT was found with gender (*p* = 0.14). However, when analysed individually, the ‘D’ component of DMFT was significantly associated with male gender (*p* = 0.05). No association of both pufa (*p* = 0.25) and PUFA (*p* = 0.29) was found with gender of the child (Table 2).

**Table 2:** Percentage prevalence of DMFT, dmft and PUFA, pufa according to child gender and age groups

Characteristics	Gender n (%)		Age groups (years)		
	Male	Female	4-5	6-12	13-17
dmft	90 (71.3)	38 (28.7)	05 (3.9)	116 (91.3)	06 (4.7)
*p* value	0.29		0.91	0.003^a^	<0.001^a^
DMFT	114 (69.9)	49 (30)	-	124 (75.1)	41 (24.8)
*p* value	0.14			0.65	0.02^a^
pufa	48 (72.7)	18 (27.2)	03 (4.5)	63 (95.4)	-
*p* value	0.25		0.86	0.11	
PUFA	48 (75)	16 (25)	-	50 (78.1)	14 (21.8)
*p* value	0.29			0.22	0.79

^a^statistically significant at 0.05 level

No PUFA value was reported for 4-5 years age group. Also, PUFA did not show significant association with children’s age groups of 6-12 years-old (*p* = 0.22) and 13-17 years-old (*p* = 0.79). Similarly, pufa values recorded in 4-5 years old (*p* = 0.86) and 6-12 years-old age (*p* = 0.11) were not significantly associated (Table 2). Although dmft did not show significant association with 4-5 years-old age group, it was found to be significantly associated with 6-12 years-old (*p* = 0.003) and 13-17 years-old age group (*p* < 0.001). The DMFT was also significantly associated with 13-17 years-old age group (*p* = 0.02). DMFT did not show any significant association with tooth brushing frequency (*p* = 0.55), tooth cleaning method used (*p* = 0.45), frequency of meals/snacks intake (*p* = 0.96) and fizzy/carbonated drinks consumption (*p* = 0.82). Similarly, dmft did not show significant association with tooth brushing frequency (*p* = 0.94), tooth cleaning method used (*p* = 1.0), frequency of meals/snacks intake (*p* = 0.90) and fizzy/carbonated drinks consumption (*p* = 0.32). No significant association was found between PUFA and tooth brushing frequency (*p* = 0.22), tooth cleaning method used (*p* = 0.68), frequency of meals/snacks intake (*p* = 0.52) and fizzy/carbonated drinks consumption (*p* = 0.54). Also, no significant association was seen for pufa and tooth brushing frequency (*p* = 0.95), tooth cleaning method used (*p* = 0.054), frequency of meals/snacks intake (*p* = 0.07) and fizzy/carbonated drinks consumption (*p* = 0.71).

Low overall caries prevalence was seen according to index ages of 5 years (2.3%), 12 years (13%) and 15 year-olds (3.8%). The 12 year-olds showed the highest overall pufa prevalence of 12.6% as compared to 5 year olds (1.7%) and 15 year-olds (1.7%) (Table 3).

**Table 3:** Prevalence of DMFT, dmft, PUFA, pufa, overall caries prevalence and overall PUFA/pufa prevalence according to WHO index ages of 5, 12 and 15 year-old children

Prevalence	5 years olds	12 year olds	15 year olds
Dmft	3.9%	11.8%	0.8%
DMFT	-	14.5%	6.1%
Overall caries prevalence	2.3%	13%	3.8%
pufa	3.1	12.3%	-
PUFA	-	15.6%	1.6%
Overall pufa prevalence	1.7%	12.6%	1.7%

The overall caries prevalence is 34.8% and overall PUFA/pufa prevalence is 15.9%. Highly significant association was found between DMFT and PUFA (*p* < 0.001) and between the orphanage and DMFT (*p* < 0.001) and PUFA (*p* = 0.02) (Table 4).

**Table 4:** Association of type of orphanage and dental visiting with DMFT/dmft and PUFA/pufa

Characteristics	DMFT n (%) (*p* value)	dmft n (%) (*p* value)	PUFA n (%) (*p* value)	pufa n (%) (*p* value)
Type of Orphanage				
Government owned	86 (52.1)	48 (37.5)	30 (46.9)	23 (34.8)
Private owned	79 (47.9)	80 (62.5)	34 (53.1)	43 (65.1)
	*p* < 0.001*	*p* = 0.43	*p* = 0.02*	*p* = 0.19
Dental visiting in past year				
Yes	46 (27.9)	32 (25)	20 (31.25)	19 (28.8)
No	119 (72.1)	96 (75)	44 (68.75)	47 (71.2)
	*p* = 0.009*	*p* = 0.21	*p* = 0.53	*p* = 0.50

*significant at 0.05 level

Dental visiting was found to be low, 28.8% for government owned and 16.6% for privately owned orphanage. More than half the children who experienced pain had not been to the dentist in the past year (66.2%) *p* = 0.013. Similarly 52.6% children who mentioned experiencing pain at night had not been to the dentist in the past year (*p* = 0.009) (Table 5).

**Table 5:** Association of type of orphanage and pain characteristics with dental visiting in past year

Characteristic	Dental visiting in past year		
	Yes	No	*p* value
Type of Orphanage			
Government owned	106 (28.8)	262 (71.2)	
Private owned	64 (16.6)	321 (83.4)	<0.001*
Do you feel any pain in your teeth			
Yes	26 (33.8)	51 (66.2)	
No	144 (21.3)	532 (78.7)	0.013*
Do you ever have pain at night			
Yes	9 (47.4)	10 (52.6)	
No	161 (21.9)	573 (78.1)	0.009*

*Significant at 0.05 level

When viewed individually, the ‘D’ component of DMFT was majorly responsible for the score of the cumulative index (*p* > 0.001) for both the orphanage type.

The results of the logistic regression analysis (Table 6) reveal that children with decay (D) were more likely to have visited the dentist (OR 3.3, 95% CI 1.42-7.6, *p* = 0.006). Also, children who had experienced pain were less likely to have visited the dentist in the past year (OR 0.53, 95% CI 0.32-0.88, *p* = 0.014).

**Table 6:** Logistic regression analysis of Decay (D) and Pain with dental visiting

Characteristic	Dental visiting *n* (%)	Odds ratio	Confidence Interval	Significance level
Decay (D)				
Absent	14 (50)			
Present	32 (23.4)	3.3	1.4-7.6	0.006^a^
Pain				
Absent	144 (21.3)			
Present	26 (33.8)	0.53	0.32-0.88	0.014^a^

^a^Significant at 0.05 level

## Discussion

This study was carried out to assess the prevalence of consequences of untreated dental decay in children residing in orphanages of the twin cities, Rawalpindi and Islamabad. This is the first study that has been undertaken in Pakistan which looked in to the clinical consequences of untreated dental decay in socially deprived children. As compared to various studies conducted in different parts of the world, low overall caries prevalence (34.8%) and low overall PUFA/pufa prevalence (15.9%) were found in our sample. Gu et al. reported 50% caries prevalence in 4-17 year old institutionalised children in China [17]. Al-Jobair et al. reported 96% caries prevalence in 4-12 year old children resident in orphanages in Saudi Arabia [16]. When DMFT/dmft and PUFA/pufa prevalence were analysed according to age groups, 24.8% DMFT prevalence and 21.8% PUFA prevalence was seen in 13-17 year-old children in our sample. Shahbong et al. reported 88.5% caries prevalence and 37.7% PUFA prevalence in 12-14 year old orphanage children in India [14]. Mehta et al. found 69.5% dmft and 38.6% pufa prevalence in 5-6 years old Indian children [23], whereas in our sample 3.9% dmft prevalence and 4.5% pufa prevalence was seen in 4-5 year-old children. On the other hand, higher dmft prevalence (91.3%) and pufa prevalence (95.4%) was seen in 6-12 year-old children in our study as compared to study reports by Thekiso et al. of 46% dmft and 41% pufa prevalence in 6-8 year-old children in South Africa [25]. Moderate level of decay was found in our sample with caries prevalence of 34.8%, however, the untreated caries ratio turned out to be 49%. This implies that almost half of the decayed teeth have progressed to pulpal involvement and abscess formation. Grund et al. report similar results of 40% caries progression into the pulp in 5 and 8 year old German children [22].

When further analysed on the basis of government or private ownership of the orphanage, it was seen that the government owned orphanage showed higher decay levels in the permanent dentition as compared to the private owned. On the other hand, PUFA score was found to be more in the private owned orphanage. Dental visiting and DMFT were found to be significantly associated, 66.2% children who felt pain in their teeth and 52.6% who felt pain at night had not been to the dentist in the past year. These results are similar to the results of study conducted in Nepal by Parai Dixit et al. which reports 93% 5-6 and 12-13 year-old children had never been to the dentist and 31% of 8-16 year-old reported having had suffered from oral pain [26]. The decay component being the major contributor of both DMFT and dmft also indicates that the dental visiting/examination pattern is problem based and not done on regular basis.

It was also seen that the ‘d’ component of dmft of primary dentition was seen most in the 10 year old age group (21.7%), when compared for all the ages (*p* < 0.001). This means that primary dentition is considered as temporary and hence not given much attention even when there is definitive need for treatment.

The symptom based dental visiting pattern is a common trend in many parts of the world and has been reported by many studies. In this regard, the pattern seen in these orphanages is no different than in children living with their families. Geottems el at reported presence of pain and not just decay was the reason for dental service utilisation by parents for their children in Brazil [27]. Another study reported symptom based dental service utilisation by both children and adolescents in Mexico [28].

Both the type of orphanages have their own faculty to provide for the educational needs of the children. How often or how much health education is imparted to the children is a concern seeing the presence of untreated decay in children. Another factor that can be responsible for the presence of decay in children is free access to sugary foods and sweetened drinks and most of the times, as a sympathetic gesture, people hand out sweets to these children which further escalates the problem.

The children had come to the orphanages at different ages and some even visit their relatives in vacations, this can also be attributed for presence of untreated decay as when children are away from the orphanage, their dietary and oral hygiene practices may differ [14].

The large sample size taken in this study provided us with an opportunity to sample children with primary and permanent dentition, hence, study decay and untreated decay levels in both the dentition. A limitation of our study is not being able to take into consideration the time period a child has spent in the orphanage or spends outside it, as this could have accounted for the influence of external environment.

## Conclusions

PUFA index can be used as an effective tool for evaluation of clinical consequences of untreated decay. When used in conjunction with DMFT index, it can provide valuable data for planning of preventive services and provision of treatment to those with urgent treatment needs. Initiation of oral health promotion programmes to train the faculty, staff and children residing in the orphanages, in oral hygiene maintenance would greatly help towards reducing the burden of oral diseases for the under privileged children.

## Additional file

Additional file 1: Dental Questionnaire. Questionnaire used to collect information from participants regarding their oral hygiene habits, dental visiting and dental pain experience. Also used to record treated and untreated decay of the participants. (DOCX 17 kb)

## Abbreviations

DMFT: Decayed, missing, filled teeth (permanent dentition)
dmft: decayed, missing, filled teeth (primary dentition)
PUFA: pulpal involvement, ulceration, fistula, abscess (permanent dentition)
pufa: pulpal involvement, ulceration, fistula, abscess (primary dentition)
UNAIDS: Joint United Nations Programme on HIV/AIDS
UNICEF: United Nations International Children’s Emergency Fund
USAID: United States Agency for International Development
WHO: World Health Organization
