# Supplementary material for: Clinical consequences of untreated dental caries assessed using PUFA index and its covariates in children residing in orphanages of Pakistan
Source: BMC Oral Health. 2017 Jul 11;17:108. doi: 10.1186/s12903-017-0399-9 (PMC5504620; doi:10.1186/s12903-017-0399-9)
Supplement: Additional file 1: — Dental Questionnaire. Questionnaire used to collect information from participants regarding their oral hygiene habits, dental visiting and dental pain experience. Also used to record treated and untreated decay of the participants. (DOCX 17 kb) [file 12903_2017_399_MOESM1_ESM.docx]

DENTAL QUESTIONNAIRE

Name: _________________ Age: _______ Sex: ________

Address: ________________ Educational Status: _________

1. How often do you brush your teeth?

□Once daily □Twice daily □Thrice daily □Never brush

2. If yes, what you use to clean the teeth?

□Tooth brush & tooth paste □Tooth brush & tooth powder

□Finger & tooth powder □Finger & salt □Neem stick

3. How many meals and snacks do you have every day?

□1 or 2 □3 or 4 □5 or more

4. How many times do you take soft/carbonated drinks?

□0-1 times/week □2-3 times/week □Almost every day

5. Have you been to a dentist past years?

□ Yes □ No

6. If yes then how often do you visit to the dentist?

□Seldom □Once a year □Twice a year or more

7. Do you have any sensitivity in your teeth?

□Yes □ No if yes, specify: _____________

8. Do you have food packing problem?

□ Yes □No if yes, specify: _____________

9. Do you feel any pain in your teeth?

□Yes □No if yes, specify: _____________

10. Do you ever have pain at night?

□Yes □No if yes, specify: _____________

11. Does any of your tooth hurt by itself?

□Yes □No if yes, specify: _____________

12. Do you have any swelling in your teeth?

□Yes □No if yes, specify: _____________

13. DMFT/dmft index:

| D | M | F | T |
| --- | --- | --- | --- |
|  |  |  |  |
| d | m | f | t |
|  |  |  |  |

14.PUFA/pufa index:

| P | U | F | A |
| --- | --- | --- | --- |
|  |  |  |  |
| p | u | f | a |
|  |  |  |  |
